# Bullous Leg Lesions Caused by *Culicoides* Midges after Travel in the Amazon Basin

**DOI:** 10.4269/ajtmh.2010.10-0236

**Published:** 2010-09

**Authors:** Ryan C. Maves, Erik J. Reaves, Gregory J. Martin

**Affiliations:** Department of Bacteriology, United States Naval Medical Research Center Detachment, Lima and Iquitos, Peru; Naval Environmental and Preventive Medicine Unit SIX, Pearl Harbor, Hawaii; Infectious Disease Clinical Research Program, Uniformed Services University, Bethesda, Maryland

**Figure 1.**
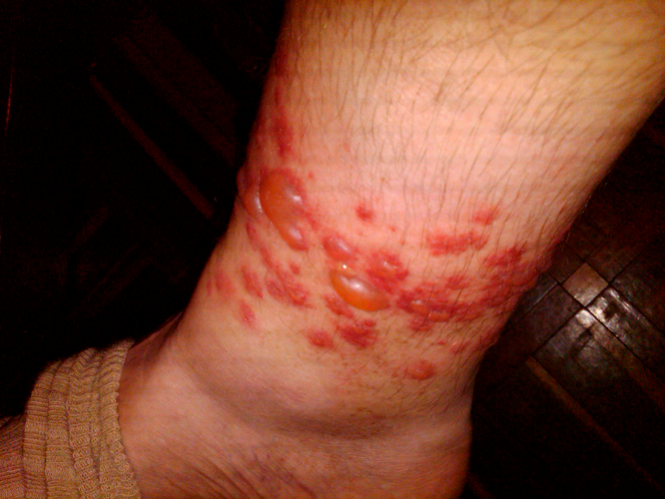
View of the patient's right ankle 1 day after travel, with prominent bullae and numerous confluent papules. Soft-tissue edema is obscuring the medial malleolus. This figure appears in color at www.ajtmh.org.

A 36-year-old man presented with 1 day of blistering lesions on the lower extremities. Two days before presentation, he had traveled to several rural communities in the Peruvian Amazon. During that trip, he had hiked through several areas of damp soil, decaying plants, and dense grass reaching up to 50 cm in height. He awoke the next morning with numerous confluent, erythematous, pruritic, non-blanching papules that were circumferential around both ankles. Over the next day, multiple bullae up to 4.5 cm in diameter developed with surrounding edema (Figure 1). The patient was treated with prednisone (50 mg daily) by mouth for 3 days, followed by routine wound care with topical antimicrobials, dressings, and topical hydrocortisone for pruritus. The lesions stabilized in size by the third day and had largely resolved by 14 days with minimal scarring.

**Figure 2.**
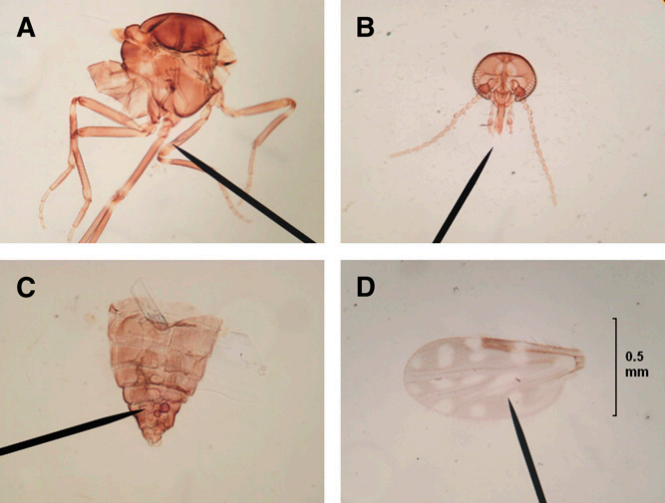
Dissecting microscope images (40×) of a female *C. paraensis* collected near Iquitos, Peru in the vicinity of where the subject traveled. (**A**) Thorax and legs. (**B**) Head. (**C**) Abdomen. (**D**) Wing. This figure appears in color at www.ajtmh.org.

This case was caused by midge bites, likely from the hematophagous species *Culicoides paraensis* and *insinuatus* (Figure 2). These small insects are widespread in the Peruvian Amazon, where they favor wet areas, grass, and decaying vegetation such as the remains of banana (platano) trees.^1^ Approximately 1 mm in size and noiseless, they are difficult to see and may be overlooked in vector-avoidance strategies despite being vectors for Oropouche virus and other pathogens. Bites to humans may manifest as an immediate-type hypersensitivity reaction with urticaria or as presumably delayed-type reactions with papular, ulcerating, or bullous lesions that can require weeks to resolve.^2^ Travelers to rural regions in the tropics should adhere carefully to vector precautions, including the use of N,N-diethyl-*meta*-toluamide (DEET), permethrin, and physical barriers such as heavy boots and tucked-in trousers to prevent bites and their potential consequences.
